# Development of genetic quality tests for good manufacturing practice-compliant induced pluripotent stem cells and their derivatives

**DOI:** 10.1038/s41598-020-60466-9

**Published:** 2020-03-03

**Authors:** Hye-Yeong Jo, Hyo-Won Han, Inuk Jung, Ji Hyeon Ju, Soon-Jung Park, Sunghwan Moon, Dongho Geum, Hyemin Kim, Han-Jin Park, Sun Kim, Glyn N. Stacey, Soo Kyung Koo, Mi-Hyun Park, Jung-Hyun Kim

**Affiliations:** 1 Division of Intractable Diseases, Center for Biomedical Sciences, Korea National Institute of Health, Cheongju, South Korea; 2 Interdisciplinary Program in Bioinformatics, Seoul National University, Seoul, South Korea; 3 Department of Computer Science and Engineering, Kyungpook National University, Buk-gu, Daegu South Korea; 4 Division of Rheumatology, Seoul St. Mary’s Hospital, College of Medicine, Catholic University of Korea, Seoul, South Korea; 5 Department of Medical Science, Konkuk University School of Medicine, Seoul, South Korea; 6 Department of Medical Science, Medical School, Korea University, Seoul, South Korea; 7 Department of Predictive Toxicology, Korea Institute of Toxicology, Daejeon, South Korea; 8 Bioinformatics Institute, Seoul National University, Seoul, South Korea; 9 Department of Computer Science and Engineering, Seoul National University, 1 Gwanak-ro, Gwanak-gu, Seoul, 08826 South Korea; 10 International Stem Cell Banking Initiative, 2 High St, Barley, Hertfordshire, SG88HZ UK; 11 National Stem Cell Resource Center, Institute of Zoology, Chinese Academy of Sciences, Beijing, 100190 China; 12 Innovation Academy for Stem Cell and Regeneration, Chinese Academy of Sciences, Beijing, 100101 China

**Keywords:** Bioinformatics, Gene expression, Induced pluripotent stem cells

## Abstract

Although human induced pluripotent stem cell (hiPSC) lines are karyotypically normal, they retain the potential for mutation in the genome. Accordingly, intensive and relevant quality controls for clinical-grade hiPSCs remain imperative. As a conceptual approach, we performed RNA-seq-based broad-range genetic quality tests on GMP-compliant human leucocyte antigen (HLA)-homozygous hiPSCs and their derivatives under postdistribution conditions to investigate whether sequencing data could provide a basis for future quality control. We found differences in the degree of single-nucleotide polymorphism (SNP) occurring in cells cultured at three collaborating institutes. However, the cells cultured at each centre showed similar trends, in which more SNPs occurred in late-passage hiPSCs than in early-passage hiPSCs after differentiation. In eSNP karyotyping analysis, none of the predicted copy number variations (CNVs) were identified, which confirmed the results of SNP chip-based CNV analysis. HLA genotyping analysis revealed that each cell line was homozygous for HLA-A, HLA-B, and DRB1 and heterozygous for HLA-DPB type. Gene expression profiling showed a similar differentiation ability of early- and late-passage hiPSCs into cardiomyocyte-like, hepatic-like, and neuronal cell types. However, time-course analysis identified five clusters showing different patterns of gene expression, which were mainly related to the immune response. In conclusion, RNA-seq analysis appears to offer an informative genetic quality testing approach for such cell types and allows the early screening of candidate hiPSC seed stocks for clinical use by facilitating safety and potential risk evaluation.

## Introduction

In the decades since the discovery of human induced pluripotent stem cells (hiPSCs) by Takahashi and Yamanaka^1^, considerable advances have been made in our understanding of these cells^2–4^. hiPSCs currently present potential clinical applications in cell therapy and regenerative medicine^5^, and with the broadening of these clinical applications, the standardization of the quality control (QC) of hiPSCs is becoming increasingly important. In particular, the evaluation of the genetic stability of the starting materials and the final product is a key consideration during QC processes for selecting suitable hiPSC lines for clinical application, as it may affect final product quality, efficacy, and safety^6,7^.

The genomes of hiPSCs are characterized by potentially wide variability, including aneuploidy, subchromosomal copy number variation (CNV), single-nucleotide variations (SNVs), and epigenetic aberrations^8^. Deletions of tumour suppressor genes and changes in immune response-related genes may not be reflected in phenotypic changes in hiPSCs; however, they could play a pivotal role once the cells have differentiated or been transplanted, although such mutations are also known to occur in perfectly healthy individuals^8^. Therefore, it may be important to perform genetic quality tests on hiPSCs and differentiated cells to facilitate the selection of hiPSC seed stocks suitable for clinical application. Accordingly, there is a global consensus regarding the need to further evaluate the genomic quality testing of cell seed stocks using procedures such as karyotyping^5,7,9,10^. However, some chromosomal abnormalities are beyond the limits of resolution of routinely used assays, which may increase the risk of missing subtle chromosomal abnormalities. Thus, it is generally agreed that more informative genomic QC tests are a prerequisite for the selection of clinical-grade hiPSCs^5,7,9,10^.

There are a wide range of technological options available for monitoring the genomic integrity of clinical-grade hiPSCs, among which single-nucleotide polymorphism (SNP) chips, fluorescence *in situ* hybridization (FISH), whole-genome sequencing (WGS), whole-exome sequencing (WES), and karyotyping are considered appropriate methodological approaches for investigating genetic alterations in seed-stock banks^6^. In addition, gene expression profiling analysis could provide better insight into the consequences of genomic alterations^8^. However, most distributors of seed-stock hiPSCs are unable to perform such comprehensive analyses using this approach, owing to cost and time limitations. Therefore, it would be highly beneficial if methods qualified specifically for the deselection of undesirable genetic variants and gene expression profiles using a single technology were available to researchers developing hiPSC lines for clinical use.

Although hiPSCs are generated and maintained based on good manufacturing practice (GMP)-compliant systems and have been approved for clinical trials, it remains to be determined which hiPSC lines are the most suitable for therapeutic application. In this regard, certain genetic variants in hiPSCs and their derivatives that are associated with human cancer, immune rejection, and cell cycle arrest could be a cause for concern, as illustrated by the decision to temporarily suspend the first clinical trial of autologous hiPSC-derived retinal cells^11^. Similarly, the impact of genetic changes on the potential of hiPSC cultures to differentiate is an important factor to be considered from a clinical application perspective. Once product safety and functional issues have been adequately addressed, an additional factor that should be considered is the human leucocyte antigen (HLA) type of the product cells to be used for allogenic transplantation. Thus, HLA-matched hiPSC lines, which could present broad applications in global and regional populations, have been suggested for use in allogenic transplantation^12,13^. Accordingly, a haplobank could provide high-quality homozygous HLA-matched hiPSC lines, thereby saving time and reducing costs while maximizing coverage^14^. Therefore, the selection of high-frequency homozygous HLA-matched hiPSCs would be beneficial; however, such selection should include the analysis of the expression of HLA molecules in hiPSC-derived products.

In this study, we investigated the utility of a single RNA-seq-based intensive genetic quality test for GMP-compliant homozygous HLA-typed hiPSC lines and their differentiated derivatives for post-distribution monitoring. Importantly, we considered the potential impact of hiPSC-based products to be used at multiple manufacturing sites^15^. Accordingly, we distributed three hiPSC lines to three separate laboratories, from which we subsequently obtained feedback, and we performed comprehensive genomic and transcriptomic profiling using samples returned by the three institutions. We found that RNA-seq-based genetic quality analysis provided a broad range of valuable information, including information on genomic variation, time-dependent changes in gene expression and HLA phenotypes. More importantly, we were able to obtain additional information using this approach to evaluate potential safety issues in the seed stock via this analytical regime.

## Results

### Scheme of the postdistribution genetic stability test for HLA-homozygous lines

**Figure 1:**
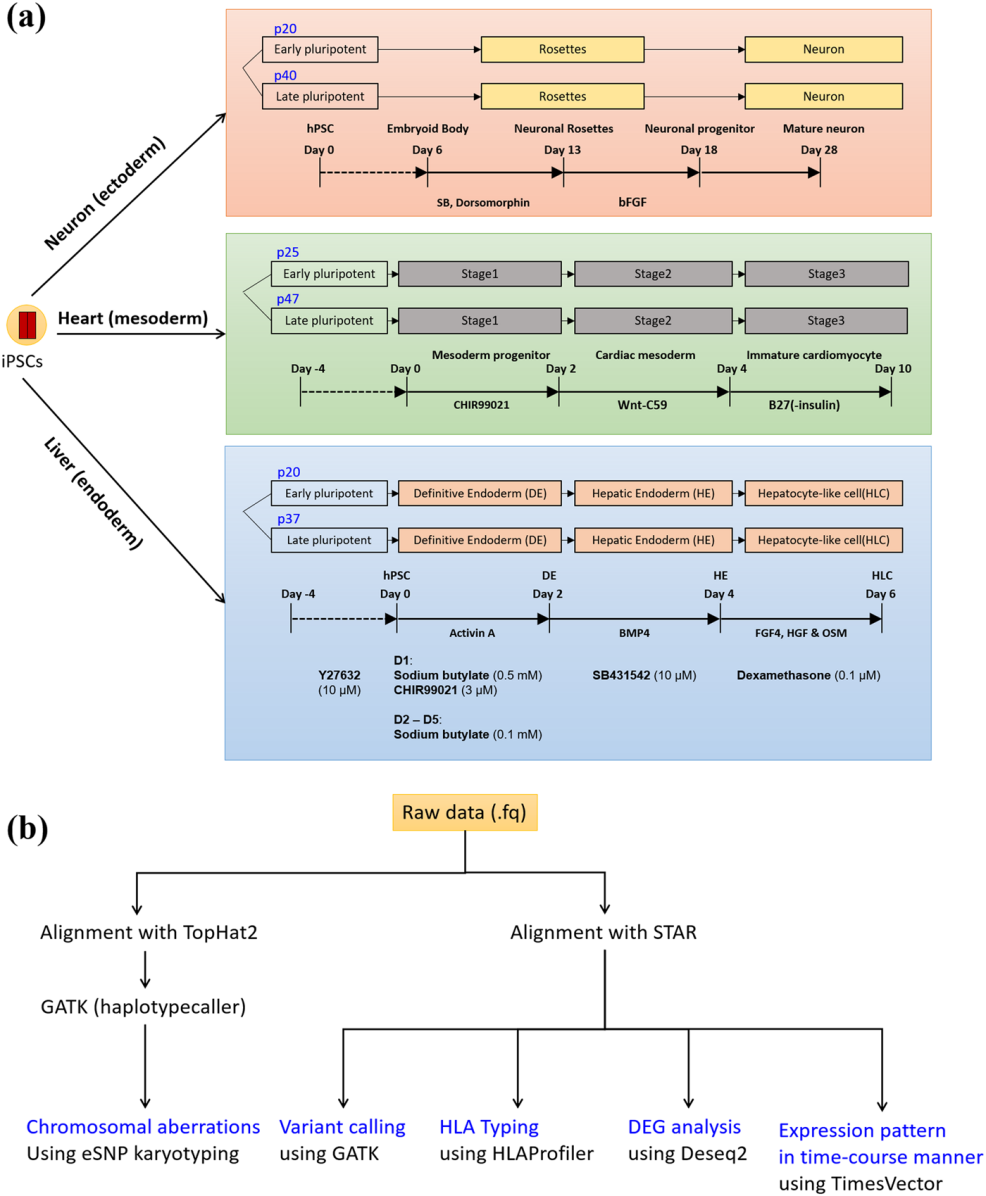
Schematic depiction of the protocol used to study the differentiation potential of good manufacturing practice-compliant human induced pluripotent stem cells (hiPSCs). (**a**) Three homozygous human leucocyte antigen (HLA)-type hiPSC lines were differentiated into three germ layers (i.e., neuronal cells, cardiomyocytes, and hepatocyte-like cells) during early and late passages at three independent collaborating institutions. The differentiation of the neuronal cells, cardiomyocytes, and hepatocyte-like cells is illustrated in orange, green, and blue boxes, respectively. The differentiation protocols are briefly presented at the bottom of each box. (**b**) Analysis workflow for the RNA-seq data of the hiPSCs and their derivatives. Five different analyses were performed. For the detection of chromosomal aberrations using eSNP karyotyping, we employed dedicated tools in the eSNP karyotyping package for alignment and variant calling.

The homozygous HLA-type GMP-compliant hiPSC lines CMC3, CMC9, and CMC11 were distributed to three external institutions, where they were expanded and differentiated into the three germ layers [neuronal cells (ectoderm), cardiomyocytes (mesoderm), and hepatocyte-like cells (endoderm); Fig. 1a]. To examine genetic stability under postdistribution conditions, differentiated cells and original hiPSC lines were collected from the three institutions and returned to the Korea Stem Cell Bank. The characteristics of the cell lines are described in Supplementary Table S1.

The workflow of the analyses performed in the present study is shown in Fig. 1b. Five different quality tests were conducted using a single RNA-seq dataset. The raw data from RNA-seq were aligned to the human reference genome using two alignment tools, TopHat2 and STAR and were subsequently subjected to genetic quality tests, including chromosomal aberration analysis, variant calling, HLA typing, DEG analysis, and time-course expression analysis. In the present study, we focused in particular on the genomic variations that may affect the genetic stability of hiPSCs and their derivatives, the significant expression levels and expression patterns of lineage-specific markers, and the effects of long-term culture.

### Differentiation into representative cells of the three germ layers

Initially, the cell lines were successfully differentiated into progenies of the three germ layers: neuronal cells, cardiomyocytes, and hepatocyte-like cells (Supplementary Figs. S1–S3), except for the CMC11 lines, which failed to undergo differentiation into hepatocyte-like cells. Among these cell lines, we performed RNA-based genomic and transcriptomic quality tests in the CMC3 hiPSC line and its derivatives, as it contains the most frequent HLA type in the Korean population.

### Identification of SNVs and indels in hiPSCs and differentiated cells

To characterize SNVs and indels on the basis of the RNAseq data, we applied a bioinformatics approach, treating the differentiated cells as the “case” and the matched hiPSCs as the “control” (Table 1). Among the total SNVs, we considered only SNVs and indels showing an alternative allelic frequency greater than 20% without missing values to retain a stringent variation dataset. In addition, we considered SNVs and indels in protein-coding regions and functional SNVs and indels, including missense variants, frameshift variants, and in-frame insertions/deletions.

**Table 1:** Single-nucleotide variants (SNVs) and indels in the differentiated lines based on RNAseq data.

No.	Layer	Control	Case	Total SNPs	>20% of alternative allelic frequency	protein_coding	functional SNPs	# of genes	#Corr. w/expression
1	**Neuron**	p20-iPS	p40-iPS	1,407	712	452	24	23	0
2		p20-iPS	p20- rosettes	1,679	850	491	20	20	0
3		p20-iPS	p20-Neuron	**1,010**	504	310	18	18	0
4		p40-iPS	p40- rosettes	1,367	699	418	16	16	0
5		p40-iPS	P40-Neuron	**1,043**	548	356	17	17	0
6	**Heart**	p25-iPS	p47-iPS	1,145	544	303	24	19	0
7		p25-iPS	p25-Stage1	1,065	441	288	7	7	0
8		p25-iPS	p25-Stage2	1,083	472	304	14	13	0
9		p25-iPS	p25-Stage3	**1,261**	659	396	20	17	0
10		p47-iPS	p47-Stage1	1,362	699	397	24	21	0
11		p47-iPS	p47-Stage2	1,014	476	304	31	28	0
12		p47-iPS	p47-Stage3	**1,671**	1,097	642	19	17	0
13	**Liver**	p20-iPS	p37-iPS	1,584	829	494	24	22	0
14		p20-iPS	p20-DE	1,198	562	358	38	37	0
15		p20-iPS	p20-HE	827	406	289	26	25	0
16		p20-iPS	P20-HLC	**1,244**	626	392	29	27	0
17		p37-iPS	p37-DE	1,616	909	572	37	34	0
18		p37-iPS	p37-HE	1,148	619	430	29	27	0
19		p37-iPS	p37-HLC	**1,509**	823	465	34	33	0

On the basis of the filtering results, we initially examined whether genes containing functional SNVs and indels affect gene expression levels, thereby enabling us to correlate gene expression at SNV and indel loci. Overall, we found that prolonged culture of hiPSCs resulted in the induction of SNVs and indels at all three participating institutes (Table 1). In addition, compared with early-passage hiPSCs, we observed that there was an increase in mutation rates when the late-passage hiPSCs were differentiated into final products (Table 1).

Next, we evaluated whether the SNVs obtained at the earlier stage of differentiation were still present at the later stage of differentiation. Indeed, 23.93–50.96% of SNVs were also detected in the last stage of cell differentiation (Supplementary Table S2). Interestingly, the number of SNVs that were maintained gradually decreased, and other mutations were produced during each stage of differentiation. In addition, approximately 40% of the protein-coding SNVs that accumulated under the prolonged culture of hiPSCs were maintained during the first stage of differentiation, and approximately 20% of these SNVs were found in terminally differentiated samples (Supplementary Table S3). However, we detected no significant differences in the expression levels of the genes retaining functional SNVs and indels, indicating that the generated SNVs and indels may not affect gene expression levels (Table 1). To determine whether the SNVs detected at the RNA level were transcribed from the DNA, we performed the whole-exome sequencing of hiPSCs, definitive endoderm (DE) cells, hepatic endoderm (HE) cells, and hepatocyte-like cells (HLCs) at passage 37 and compared the results to the RNA-seq data. Among the maintained SNVs identified via RNA-seq analysis, approximately 34.26–50.30% of the SNVs were transcribed from the DNA during hepatocyte differentiation (Supplementary Table S2).

To investigate SNVs at the genomic level, we performed a SNP chip (Cytoscan HD array, Affymetrix)-based SNV analysis (Supplementary Table S4). Although we identified several nonsynonymous SNVs, we detected virtually no correlations between the final SNVs and gene expression levels (threshold: 2-fold change) in any of the comparisons; the one exception was the identification of significant heterozygous SNVs in the *PRDM14* gene at an early passage in stage three of cardiomyocyte differentiation that may result in the downregulation of gene expression (Supplementary Table S4). However, all of the other differentiated cells that we examined in the present study showed lower expression of *PRDM14* compared with hiPSCs devoid of SNVs (Supplementary Fig. S4), indicating that the differences in *PRDM14* gene expression may not reflect SNVs. Thus, SNV analyses using both SNP chip and RNAseq data revealed the accumulation of nonsynonymous SNVs and indels during the prolonged culture and differentiation of hiPSCs, although these variants showed no significant effects on gene expression.

### Evaluation of chromosomal aberrations of hiPSCs and differentiated cells

**Figure 2:**
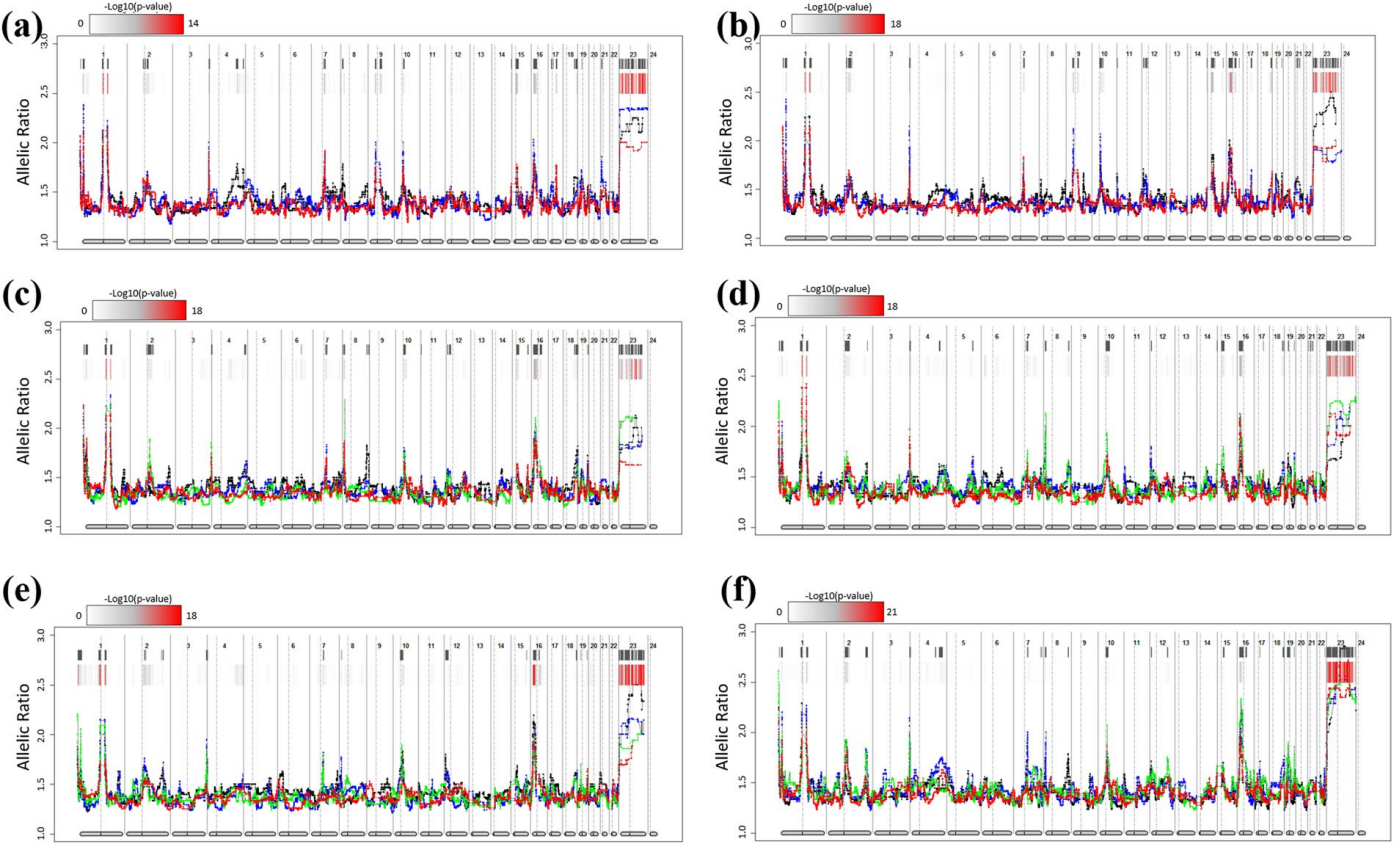
Detection of chromosomal aberrations in human induced pluripotent stem cells (hiPSCs) and their derivatives using eSNP karyotyping. Moving median plots of 151 single-nucleotide polymorphisms (SNPs) of expressed genes from the RNA-seq data of all 22 cell lines used in this study are shown. For neuronal cells, hiPSCs and their neuronal derivatives are shown in a single plot at early (**a**) and late (**b**) passages, respectively. Black, red, and blue in the plot indicate hiPSCs, rosette progenitors, and neuronal cells, respectively. For cardiomyocytes, hiPSCs and their cardiomyocyte derivatives are shown in a single plot at early (**c**) and late (**d**) passages, respectively. Black, blue, green, and red in the plot indicate hiPSCs, stage 1 differentiation, stage 2 differentiation, and stage 3 differentiation, respectively. For hepatocytes, hiPSCs and their hepatocyte derivatives are also shown in a single plot at early (**e**) and late (**f**) passages, respectively. Black, blue, green and red in the plot indicate hiPSCs, definitive endoderm (DE) cells, hepatic endoderm (HE) cells, and hepatocyte-like cells (HLC), respectively. Coloured bars represent FDR-corrected p-values. Positions with p-values < 0.01 are indicated with a black line.

It has been suggested that chromosomal aberrations that occur during the differentiation of hiPSCs may have unexpected consequences with respect to the propensity for differentiation and, ultimately, regenerative medicine^16^. We examined chromosomal aberrations in hiPSCs and their derivatives based on RNA-seq data using eSNP-karyoryping^17^, measuring the ratio of expression between the two alleles (Fig. 2). The eSNP-karyotyping of all of the lines revealed that the lines presented normal diploid karyotypes. We also found that the major/minor SNP ratios were similar between hiPSCs and their differentiated derivatives, indicating that no chromosomal aberrations had occurred during either the early or late passages.

Using SNP chip genotyping data, we also analysed CNV changes in the differentiated lines in the final stages compared with the original cell line at the genome-wide level. We found no significant differences in CNVs between the hiPSCs and the derivative cell lines under prolonged culture at the whole-genome level. Notably, we detected no CNVs in the 1q41, 12p13.31, 17q25.2, and 20q11.21 regions (which have been reported to affect the genomic stability of hiPSCs^9,18,19^) in the homozygous HLA cell lines and derivative cells (Supplementary Fig. S5a–d).

### Evaluation of cancer-related gene expression

To investigate whether the differentiated lines showed significant genomic instability in terms of tumourigenicity compared with the original lines during early and late passages, we sought to identify DEGs among cancer-related genes between the hiPSCs and differentiated cells showing significant expression changes. Among the 707 candidates^20,21^, we identified a number of genes exhibiting significant changes in expression during the differentiation process. In neuronal cells, the cancer-related gene *SLIT2* was found to be upregulated (Supplementary Fig. S5e), as were six other cancer-related genes (*PDGFRA*, *DDR2*, *RSPO3*, *IL6ST*, *NTRK2*, and *HEY1*) in cardiomyocytes (Supplementary Fig. S5f) and one cancer-related gene (*HSD3B1*) in hepatocyte-like cells (Supplementary Fig. S5g). However, in hiPSCs, the expression levels of these tumourigenicity-associated genes were similar during differentiation, regardless of the number of passages. These changes in gene expression were found to be consistent in replicates of differentiated cultures.

### Quality testing of HLA types in differentiated cells using RNA-seq data

To determine the changes in HLA types during the differentiation or passaging of cells, we subsequently investigated the expression of HLA molecules in both the hiPSCs and their differentiated derivatives for QC testing using RNA-seq data (Table 2 and Supplementary Table S5). We specifically focused on major *HLA* genes, including both MHC class I (*HLA-A*, *HLA-B*, *HLA-C*, *HLA-E*, and *HLA-F*) and MHC class II (*HLA-DPA1, HLA-DPB1, HLA-DPB2*, *HLA-DQB1*, *HLA-DRB1*, and *HLA-DRB3*) genes that are known to cause allogenic immune rejection in clinical settings. With the exception of *HLA-DPB1*, we found that both the hiPSCs and differentiated lines showed homozygous HLA types for all the detectable HLA molecules analysed using HLAProfiler. In contrast, we detected a heterozygous HLA type in the case of HLA-DPB1.

**Table 2:** Human leucocyte antigen (HLA) types of human induced pluripotent stem cells (hiPSCs) and differentiated cells.

Cell Line		A		B		E		DRB1		DPB1	
		Allele1	Allele2	Allele1	Allele2	Allele1	Allele2	Allele1	Allele2	Allele1	Allele2
Neuronal Cells	iPS_p20	A*33:03:01	A*33:03:01	*n.d*	*n.d*.	E*01:03:01:01	E*01:03:01:01	*n.d*	*n.d*.	DPB1*02:02	DPB1*04:01:01:01
	Rosettes_p20	A*33:03:01	A*33:03:01	B*44:03:01:01	B*44:03:01:01	E*01:03:01:01	E*01:03:01:01	*n.d*.	*n.d*.	DPB1*04:01:01:01	DPB1*02:02
	Neuronal Cells_p20	A*33:03:01	A*33:03:01	*n.d*.	*n.d*.	E*01:03:01:01	E*01:03:01:01	*n.d*.	*n.d*.	DPB1*02:02	DPB1*04:01:01:01
	iPS_p40	A*33:03:01	A*33:03:01	B*44:03:01:01	B*44:03:01:01	E*01:03:01:01	E*01:03:01:01	*n.d*.	*n.d*.	DPB1*02:02	DPB1*04:01:01:01
	Rosettes_p40	A*33:03:01	A*33:03:01	B*44:03:01:01	B*44:03:01:01	E*01:03:01:01	E*01:03:01:01	*n.d*.	*n.d*.	DPB1*02:02	DPB1*04:01:01:01
	Neuronal Cells_p40	A*33:03:01	A*33:03:01	B*44:03:01:01	B*44:03:01:01	E*01:03:01:01	E*01:03:01:01	*n.d*.	*n.d*.	DPB1*02:02	DPB1*04:01:01:01
Cardio-myocytes	iPS_p25	A*33:03:01	A*33:03:01	B*44:03:01:01	B*44:03:01:01	E*01:03:01:01	E*01:03:01:01	*n.d*.	*n.d*.	DPB1*02:02	DPB1*04:01:01:01
	stage1_p25	A*33:03:01	A*33:03:01	B*44:03:01:01	B*44:03:01:01	E*01:03:01:01	E*01:03:01:01	*n.d*.	*n.d*.	DPB1*02:02	DPB1*04:01:01:01
	stage2_p25	A*33:03:01	A*33:03:01	*n.d*.	*n.d*.	E*01:03:01:01	E*01:03:01:01	*n.d*.	*n.d*.	DPB1*02:02	DPB1*04:01:01:01
	stage3_p25	A*33:03:01	A*33:03:01	*n.d*.	*n.d*.	E*01:03:01:01	E*01:03:01:01	*n.d*.	*n.d*.	DPB1*02:02	DPB1*04:01:01:01
	iPS_p47	A*33:03:01	A*33:03:01	*n.d*.	*n.d*.	E*01:03:01:01	E*01:03:01:01	*n.d*.	*n.d*.	DPB1*02:02	DPB1*04:01:01:01
	stage1_p47	A*33:03:01	A*33:03:01	*n.d*.	*n.d*.	E*01:03:01:01	E*01:03:01:01	*n.d*.	*n.d*.	DPB1*02:02	DPB1*04:01:01:01
	stage2_p47	A*33:03:01	A*33:03:01	B*44:03:01:01	B*44:03:01:01	E*01:03:01:01	E*01:03:01:01	*n.d*.	*n.d*.	DPB1*02:02	DPB1*04:01:01:01
	stage3_p47	A*33:03:01	A*33:03:01	B*44:03:01:01	B*44:03:01:01	E*01:03:01:01	E*01:03:01:01	*n.d*.	*n.d*.	DPB1*02:02	DPB1*04:01:01:01
Hepato-cytes	iPS_p20	A*33:03:01	A*33:03:01	*n.d*.	*n.d*.	E*01:03:01:01	E*01:03:01:01	*n.d*.	*n.d*.	DPB1*02:02	DPB1*04:01:01:01
	DE_p20	A*33:03:01	A*33:03:01	B*44:03:01:01	B*44:03:01:01	E*01:03:01:01	E*01:03:01:01	DRB1*13:02:01	DRB1*13:02:01	DPB1*02:02	DPB1*04:01:01:01
	HE_p20	A*33:03:01	A*33:03:01	*n.d*.	*n.d*.	E*01:03:01:01	E*01:03:01:01	DRB1*13:02:01	DRB1*13:02:01	DPB1*02:02	DPB1*04:01:01:01
	HLC_p20	A*33:03:01	A*33:03:01	B*44:03:01:01	B*44:03:01:01	E*01:03:01:01	E*01:03:01:01	*n.d*.	*n.d*.	DPB1*02:02	DPB1*04:01:01:01
	iPS_p37	A*33:03:01	A*33:03:01	B*44:03:01:01	B*44:03:01:01	E*01:03:01:01	E*01:03:01:01	*n.d*.	*n.d*.	DPB1*02:02	DPB1*04:01:01:01
	DE_p37	A*33:03:01	A*33:03:01	B*44:03:01:01	B*44:03:01:01	E*01:03:01:01	E*01:03:01:01	DRB1*13:02:01	DRB1*13:02:01	DPB1*02:02	DPB1*04:01:01:01
	HE_p37	A*33:03:01	A*33:03:01	B*44:03:01:01	B*44:03:01:01	E*01:03:01:01	E*01:03:01:01	DRB1*13:02:01	DRB1*13:02:01	DPB1*02:02	DPB1*04:01:01:01
	HLC_p37	*n.d*.	*n.d*.	B*44:03:01:01	B*44:03:01:01	E*01:03:01:01	E*01:03:01:01	*n.d*.	*n.d*.	DPB1*02:02	DPB1*04:01:01:01

N. D.: not determined.

Importantly, the homozygous HLA types were maintained upon differentiation from the initiating hiPSCs during both early and late passages. We also found that class I MHC molecules showed increased expression compared with MHC class II molecules and that only the DPB2 type was detected in hiPSCs during both early and late passages (Supplementary Table S5). It should be noted here that HLA typing was not carried out in those cases in which there were fewer than 100 reads for *HLA*s.

### Transcriptome profiling revealing effective differentiation into the three germ layers

**Figure 3:**
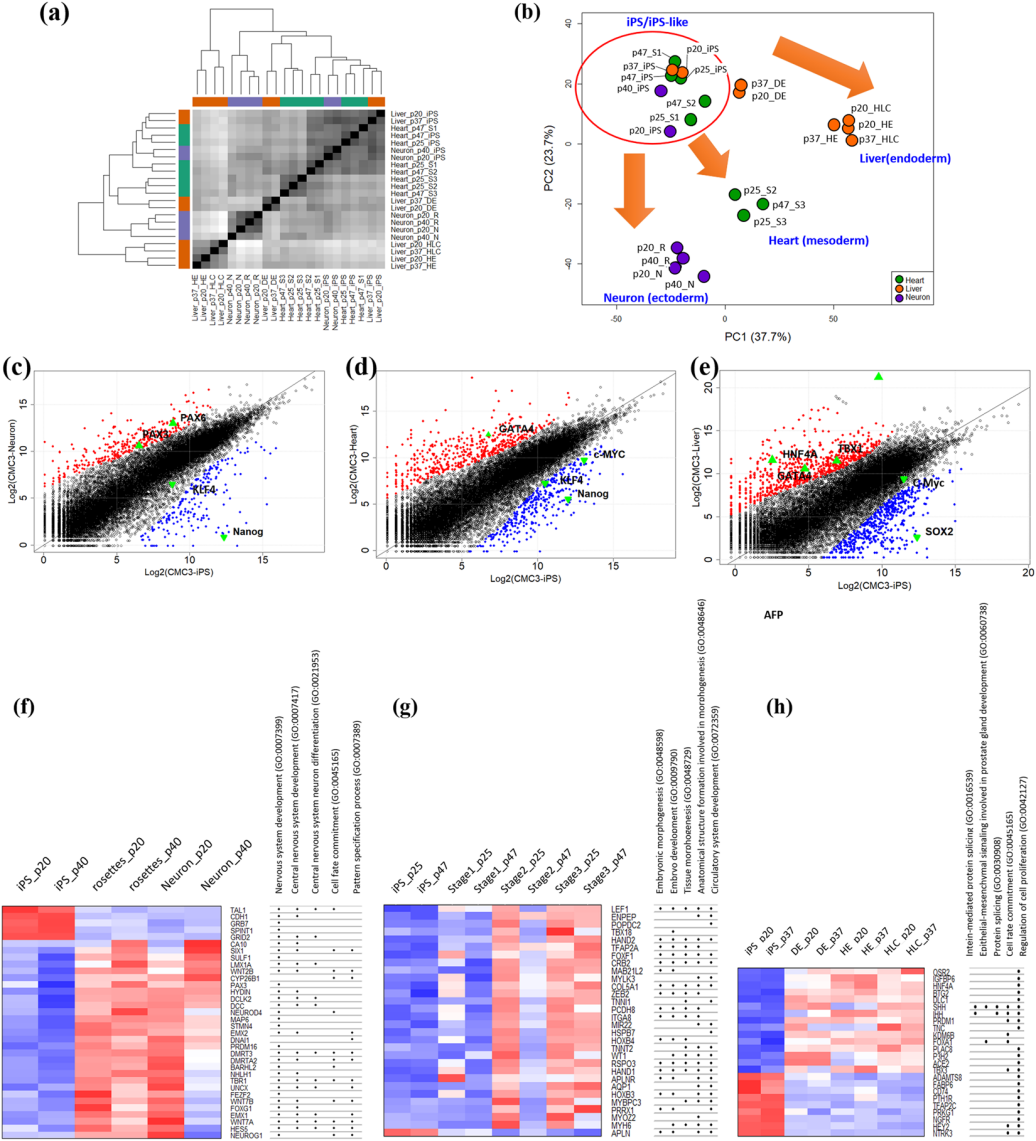
Characterization of differentiated cells of three lineages and the original human induced pluripotent stem cells (hiPSCs) at the transcriptome level. Global transcriptome analysis of hiPSC lines and differentiated cells. (**a**) Relationship of transcriptome profiles among the cells. Sample-to-sample distance matrix with hierarchical clustering. (**b**) Principal component analysis (PCA) of all lines. Neuronal cells (purple circles), cardiomyocytes (green circles), and hepatocyte-like cells (orange circles) were differentiated from hiPSCs. hiPSCs and iPS-like cells that divided with hiPSCs are highlighted with red circles. (**c**–**e**) Scatter plot of log2-normalized read counts for differentiated lines, neuronal cells, cardiomyocytes, and hepatocytes in the final stages compared with the original hiPSC lines. Lineage-specific markers for each lineage (filled triangles) and hiPSC-specific markers (inverted filled triangles) are indicated. Up- and downregulated genes are also denoted by red and blue circles, respectively. PAX3, paired box 3; PAX6, paired box 6; KLF, Krüppel-like factor 4; GATA binding protein 4, GATA4; AFP, alpha foetoprotein; TBX1, T-box 1. The top five gene ontology (GO) categories for the common differentially expressed genes (DEGs) identified during the differentiation process for each lineage (i.e., neuronal cells (**f**), cardiomyocytes (**g**), and hepatocyte-like cells (**h**)) are shown. The heatmap for GO analysis indicates the expression of each gene at each stage during differentiation.

To evaluate the differentiation potential of early- and late-passage homozygous HLA-typed hiPSC lines, we carried out global gene expression profiling at the transcriptome level. We initially analysed the genetic distances between all of the samples examined in this study (Fig. 3a). Heatmap-based hierarchical clustering separated the hiPSC lines from the three lineages (neuronal cells, cardiomyocytes, and hepatocyte-like cells). In addition, the cells tended to be closely clustered according to their differentiation stage. One exception in this regard was found for stage 1 cells in the cardiomyocyte lineage at a late passage (passage 47), which showed closer clustering with the hiPSC line, thereby indicating the retention of hiPSC-like characteristics.

PCA revealed four groups of cells showing distinct expression patterns, which could be classified as hiPSCs/hiPSC-like cells, neuronal cells, cardiomyocytes, and hepatocyte-like cells (Fig. 3b). PC1 and PC2 captured 37.7% and 23.7% of the variability in gene expression, respectively. The hiPSCs and each cell lineage branched in different directions corresponding to the four different cell types. In neuronal cells, the changes in the expression patterns observed during differentiation from hiPSCs to neurons via rosette progenitor and neuronal cells were clustered: the changes between hiPSCs and stage 3 cardiomyocyte differentiation showed that stage 1 and stage 2 cardiomyocytes were more similar to hiPSCs, in accord with the results of the sample distance matrix (Fig. 3a and b). In hepatocyte-like cells, we identified changes in expression patterns during differentiation from hiPSCs to HE and HLC via DE cells during both early and late passages, consistent with the results presented in Fig. 3a.

We also identified changes in gene expression for lineage-specific markers during the final stages of differentiation compared with the respective undifferentiated cell lines. In neuronal cells, neuronal cell-specific markers, such as PAX3 and PAX6, were upregulated, whereas self-renewal markers, such as KLF4 and Nanog, were downregulated (Fig. 3c). In cardiomyocytes, the cardiomyocyte-specific marker GATA4 was upregulated, whereas the self-renewal markers Nanog, c-Myc, and KLF4 were downregulated (Fig. 3d). Furthermore, in hepatocyte-like cells, hepatocyte-specific markers such as HNF4α, AFP, GATA4, and TBX1 were upregulated, whereas self-renewal markers such as SOX2 and c-Myc were downregulated (Fig. 3e). These results thus indicated that in each lineage, the final stage of the differentiation process is associated with lineage-specific characteristics at the transcriptome level, suggesting that the CMC3 HLA-homozygous hiPSC line exhibits a stable differentiation potential for the three lineages, regardless of passaging.

We subsequently focused on a specific set of DEGs that were maintained at significant levels from the mid-stage to the final stage of differentiation when the hiPSCs had differentiated into each layer, as they could be lineage-specific genes (Supplementary Tables S6–S8). GO analysis was performed to determine the functional roles of these genes in biological processes. In neuronal cells, we found that 104 DEGs were enriched in nervous system development, central nervous system development, central nervous system neuronal cell differentiation, cell fate commitment, and pattern-specific processes (Fig. 3f), indicating that these genes are related to neuronal development. In cardiomyocytes, the top-ranked GO categories for the 82 DEGs identified during cardiomyocyte differentiation were related to development, including embryo development, tissue morphogenesis, anatomical structure formation involved in morphogenesis, and circulatory system development (Fig. 3g). Similarly, the top-ranked GO categories for the 99 DEGs related to hepatocyte-like cell differentiation were enriched in intein-mediated protein splicing, epithelial-mesenchymal signalling involved in prostate gland development, cell fate commitment, and the regulation of cell proliferation (Fig. 3h).

### Time-course transcriptome analysis to evaluate the effects of the prolonged culture of hiPSCs on the differentiation process

**Figure 4:**
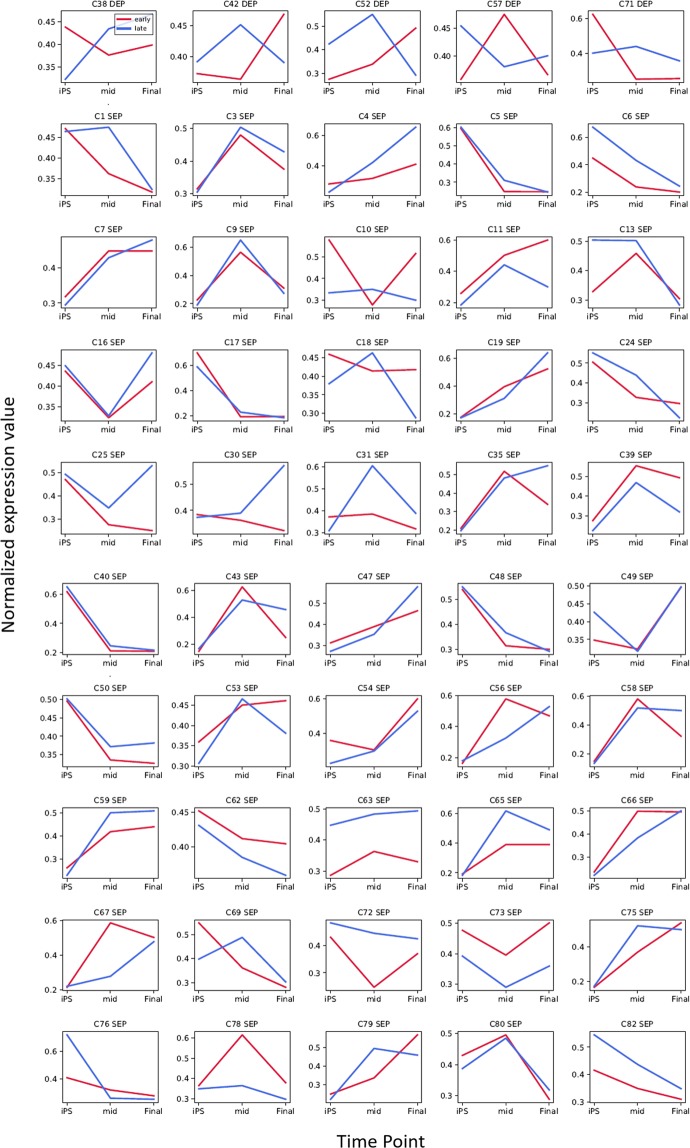
Time-course transcriptome analysis of differentiation during early and late passages. Comparison of expression profiles between early (red bars) and late (blue bars) passages at three time points [human induced pluripotent stem cells **(**hiPSCs), the middle stage of differentiation, and the final stage of differentiation]. Forty similar expression patterns (SEPs) and five differential expression profiles (DEPs) are shown in the top and bottom panels, respectively. The normalized expression values were applied to calculate relative expression. The number at the top of each graph indicates the number of clusters. ODEP: obscure DEP.

**Figure 5:**
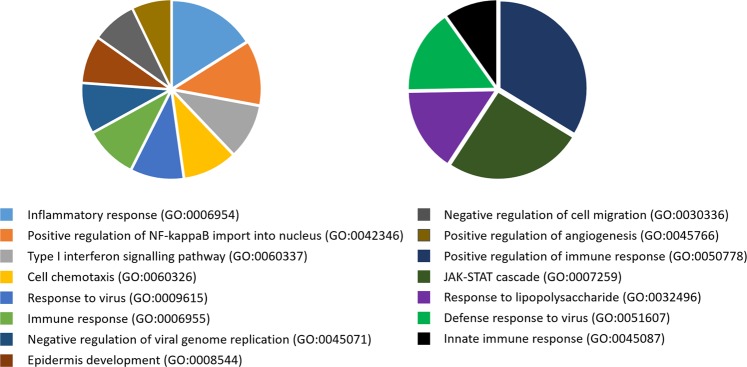
Gene ontology enrichment analysis of the genes of differential expression profiles (DEPs). Pie charts show the top-ranked GO terms of biological processes for the genes in Cluster 38 (left) and cluster 72 (right). The more significant the GO term, the larger the portion of the pie chart. Only GO terms with a p-value < 0.05 were considered.

In the present study, we were particularly interested in the ‘passaging’ effect on the original cells, with a view towards categorizing differentiation into the three germ layers at sequential stages. To this end, we performed a time-course transcriptome analysis comparing early (passages 20–25) and late (passages 37–47) passages of hiPSCs during differentiation at three time points to predict phenotypic changes under prolonged culture, although there were no significant genetic abnormalities detected. Among the significant gene clusters, we detected 45 types of similarly expressed profiles (SEPs) (Fig. 4), indicating that the early- and late-passage hiPSCs differentiated in a similar pattern overall. However, five types of differential expression profiles (DEPs) were detected. We subsequently performed GO enrichment analysis to determine the functional roles of the genes in the DEP clusters. GO analyses of the genes in cluster 38 (C38) and cluster 71 (C71) showed significant enrichment in biological processes, among which the most significantly enriched terms (p-value < 0.05) are presented in Fig. 5 (Supplementary Table S9). Interestingly, the top-ranked GO terms were associated with immunogenicity, such as the inflammatory response, the type I interferon signalling pathway and the immune response, suggesting that ‘passaging’ may have certain immunogenicity-related repercussions on hiPSCs and their differentiation propensity.

## Discussion

hiPSCs can undergo certain genomic changes during the course of proliferation and differentiation^7,9^. Consequently, to minimize any potentially adverse effects of acquired aberrations on cell therapies involving hiPSCs and their derivatives, it may be valuable to monitor hiPSC cultures throughout the preparation process using high-resolution techniques, preferably in a cost-effective manner^22^.

In this study, we performed SNV, indel, CNV, ekaryotyping, HLA genotyping and transcriptomic analyses in which differentiated cells were compared with hiPSCs using RNA-seq data from undifferentiated and differentiated cultures returned from multiple centres. In particular, we evaluated whether prolonged culture conditions have the potential to give rise to genomic and phenotypic changes in the CMC3 hiPSC line, a candidate line for manufacturing cell therapies. RNA-seq data analysis revealed that when the CMC3 hiPSC line was subjected to prolonged culturing, these cells developed a greater number of SNVs in samples from all three collaborators. However, we detected no significant correlations between SNVs and the observed changes in gene expression. Furthermore, none of the predicted CNVs were detected in either the RNA-seq-based ekaryotyping or SNP chip-based analyses.

RNA-seq-based genetic stability testing showed that GMP-compliant CMC3 hiPSCs and their derivatives contained none of the predicted CNVs or important SNVs. In addition, no tumourigenic potential was identified, indicating that the hiPSC line could be safely applied in the clinical setting. However, the efficacy of the cell line in QC regimes, particularly as a clinical-grade seed stock that will be distributed and applied in diverse clinical settings for cell therapy, should be considered. When we examined phenotypic changes using gene expression profiling, we found that early- and late-passage CMC3 hiPSCs showed similar capacities to differentiate into cardiomyocytes, hepatic-like cells, and neuronal cells. In addition, RNA-seq-based efficacy testing results were obtained in phenotypic assays. These data indicated that the parameters of RNA-seq-based analysis were useful for cell line efficacy testing to reduce costs and save time in product development. Based on these safety and efficacy-related QC parameters and the results for the CMC3 line, we could consider the distribution of this line for clinical application.

We highlight that RNA-seq can facilitate in-depth genomic and transcriptomic analyses that can provide valuable insights into the consistency of the quality and integrity of hiPSC stocks and products under multisite manufacturing conditions, as needed to introduce these advanced therapies worldwide. However, there are some limitations to RNA-seq-based genomic and transcriptomic data analysis. First, transcript sequences are not completely faithful to the corresponding DNA sequences. Owing to RNA-DNA differences, we may have missed some important genetic abnormalities in the seed stocks^23^. Second, sequences showing low expression could not be evaluated. In particular, some genes showing high expression in hiPSC stocks were not expressed in differentiated cells, and it was therefore difficult to track their mutation patterns throughout the differentiation process.

We found that SNVs in the CMC3 hiPSC line generally had no significant effect on gene expression during passaging and differentiation. One exception was presented by a significant SNV detected in the *PRDM14* gene, which was downregulated in stage 3 of cardiomyocyte differentiation from early-passage CMC3 hiPSCs. *PRDM14* plays important roles in maintaining stemness and self-renewal in embryonic stem cells through epigenetic mechanisms^24^. Although further studies are needed to determine whether the *PRDM14* mutation directly alters gene expression, we found in the present study that all differentiated cells showed lower expression of *PRDM14* than hiPSCs. Therefore, it is conceivable that the downregulation of PRDM14 expression may be associated with the process of differentiation rather than being a consequence of SNVs in the PRDM14 sequence. Accordingly, this interpretation serves to highlight the importance of intensive analysis to identify mutations causing changes in gene expression that could affect the quality and safety of hiPSC-derived therapeutic products.

The analysis of tumourigenic potential is essential to assure the quality of hiPSC products, particularly with respect to their potential efficacy and safety in clinical applications^25^. In the present study, we evaluated changes in the expression of previously reported cancer-associated genes in hiPSCs and each derived lineage. Although prolonged culture did not appear to affect the expression of cancer-related genes, we found that several genes underwent passage-related changes during the differentiation of hiPSCs. These genes play diverse roles, including the suppression of tumours and the promotion of normal differentiation. However, when we closely examined the functions of the DEGs, we found that most of the genes were involved in the suppression of cancer progression or were related to pluripotent stem cells and cell differentiation (Supplementary Fig. S5e-f). For example, *SLIT2*, which is known to suppress tumour progression and metastasis^26^, showed increased expression during neuronal cell differentiation. In contrast, we found that Sox2, a pluripotency marker that plays an important role in tumour development and cancer proliferation^27^, showed decreased expression during cardiomyocyte differentiation. Similarly, LCK, which is overexpressed in colon and lymphoma cancer^28^, was downregulated in cardiomyocyte cells compared with hiPSCs. We note that these cancer-related genes are also associated with lineage-specific differentiation^29–31^. However, we identified one gene for which changes in expression could pose a potential risk: the CDH1 gene. We found that compared with hiPSCs, this tumour suppressor gene was downregulated in differentiated neuronal cells. Although CDH1 also plays an important role in pluripotent stem cell self-renewal and may therefore be downregulated during the differentiation process, this protein poses a potential cancer-related risk if expressed in the final therapeutic cell population because its expression indicates the presence of stem cells capable of producing benign but proliferating tumours. Therefore, it is necessary to assess changes in cancer-related genes during differentiation in more detail, including the application of *in vivo* tumourigenesis assays, which will be crucial for any hiPSC-based product release^10^.

Given that transplanted allogenic hiPSCs and their products can be immune-rejected by allogenic and autologous natural killer cells^32^, it is imperative that we monitor the major HLA types of hiPSCs and their derivatives to prevent adverse immune responses after the transplantation of their differentiated progeny. As a QC step, most haplobanks conduct tests for major HLA types, including HLA-A, HLA-B, and HLA-DR, via polymerase chain reaction (PCR). Nevertheless, there is still a risk of mutations in other unmonitored HLA genes during differentiation and long-term passaging, and appropriate methods for quality testing therefore need to be developed for haplobanks. In the present study, we demonstrated the expression of HLA and the lack of mutations in the *HLA* locus in a seed stock and its derivatives for the first time. Due to cost and time limitations, donor compatibility tends only to be evaluated with respect to major HLA types; however, the importance of other HLA types in immune responses has been reported and should therefore not be overlooked^33^. For example, HLA-E-expressing hiPSCs can be immune-tolerant by avoiding potential allogenic responses^34^. Notably, the RNA-seq-based genotyping approach used in this study provided valuable information characterizing the expression of HLA molecular types that are not normally targeted in genotyping, such as HLA-E, HLA-F, DPA1, DPB2, and DQB1. In the CMC3 seed stock, we identified homozygous HLA-E, HLA-F, DPA1, DPB2, and DQB1 types, which were maintained during differentiation. However, RNA-seq-based QC is unable to detect the haplotypes of weakly expressed *HLA* genes, and in some cases, we found that the HLA types showed expression below the detectable level, which this could be a limitation of RNA-seq-based QC analysis. Therefore, important HLA types with lower expression may require PCR-based QC tests.

Time-course transcriptional analysis allows the investigation of transcriptional regulatory networks and provides information regarding the dynamic behaviour of the genes associated with different phenotypes. This approach is particularly useful for the identification of the differential coexpression of biomarkers that are involved in the same biological processes, providing insights into the dynamics of their transcriptional activity under certain conditions^35^. Despite showing a normal karyotype, genetic aberrations tend to manifest in hiPSCs after an extended time in culture, giving rise to ‘culture-adapted’ and more rapidly growing hiPSCs^9^, which can influence the propensity of these cells to differentiate^36,37^. To explore this issue, we applied a conceptual time-course transcriptional analysis to prolong the passaging of hiPSCs and compared the expression profiles of hiPSCs between early and late passages at three time points (hiPSCs, the middle stage of differentiation, and the final stage of differentiation). The results revealed that whereas the expression profiles of early- and late-passage cells were, for the most part, similar, five clusters showed different patterns. More importantly, these clusters comprised immune response-related genes, which are considered to pose a significant risk in cell therapy; specifically, the immunogenicity of an hiPSC-derived product in autologous and allogenic human immune systems could cause a T-cell immune response.

The time-course transcriptomic analysis data provided valuable information regarding the safety of the product derived from the prolonged passaging of hiPSCs. However, more data from multiple clinical-grade homozygous hiPSC lines from multiple centres are required to validate our assay. To collect such extended datasets, we suggest the global networking of clinical-grade hiPSC banking entities to facilitate the development of better critical quality assessment (CQA) strategies for seed stocks.

The identification of lineage-specific differentiation markers and markers of undifferentiated cells is a critical step in the clinical application of PSC-derived cell therapy, as such markers could present applications in CQA and should be measured and monitored during manufacturing. Therefore, researchers have investigated PSC markers and tissue-specific differentiation markers using various techniques^38^. In this study, we performed a systematic time-course transcriptome analysis to identify lineage-specific genes during differentiation. In total, we identified 70, 126, and 107 genes in neuronal cells, cardiomyocytes, and hepatocyte-like cells, respectively (Supplementary Fig. S6 and Supplementary Table S10), most of which (with the exceptions of the cardiomyocyte-specific gene *HAND1* and the hepatocyte-specific gene *ALB*) have not previously been identified as specific differentiation markers. In addition, we analysed hiPSC-specific gene clusters showing decreasing gene expression in all lineages. Our findings indicate that *EMX2OS*, *EMX2*, *DMRT3*, *C1orf61*, and *TBR1* can serve as specific markers of neuronal cells, whereas *HAND2*, *AQP1*, *HAND1*, *ITGA8*, and *MYH6* can be considered cardiomyocyte-specific markers. These candidates play essential roles in the development of ectoderm and mesoderm, respectively. Unlike lineage-specific markers, we also found a gene cluster that showed continuously decreased expression during differentiation in all lineages (i.e., a non-specific gene cluster). This cluster, which comprised 134 genes, may be a hiPSC-specific gene cluster or a cluster that affects the genomic stability of hiPSCs and their differentiated lines and included the known stemness-related markers *POU5F1* and *NANOG* (Supplementary Table S10). Although further studies will be necessary to confirm the functional effects of the lineage-specific or stemness-related genes identified in the present study, the data presented here provide important insights for those working in the fields of bioinformatics and systems biology research on hiPSCs.

The development of suitable QC parameters and methods for evaluating genetic stability in clinical-grade seed-stock banking is not yet standardized. A global consortium of expert stem cell researchers has concluded that the development of genetic variants found in hPSCs is a critical step for understanding the safety implications of advanced hPSC-derived products; therefore, the assessment of genetic integrity may be most critical for the final product^18,39^. At this stage of cell therapy development, the collection of genetic stability data alongside product manufacturing is valuable not only as part of QC or release testing but also for future utilization to collate clinical outcomes and patient follow-up. Therefore, we believe that this database could contribute to the exploration of the utility of developing routine QC and risk assessment procedures^10,40^.

## Methods

### Differentiation into the three germ layers

The hiPSC lines were expanded and differentiated into neuronal cells, cardiomyocytes, and hepatocyte-like cells. For neuronal cell differentiation, hiPSCs were initially differentiated into neural ectoderm via embryoid body formation using STEMdiff Neural Induction Medium (NIM; cat. no. 05835; Stem Cell Technologies) and SB431542 (1614; Tocris) and Dorsomorphin (Ab144821; Abcam) at 3 μM until they had attained a size of approximately 1 mm in diameter. Subsequently, the embryoid bodies were transferred to plates coated with Matrigel (354277; Corning), and neural rosette formation was induced using NIM. Thereafter, the neuronal rosettes were further differentiated into neuronal precursor cells using bFGF (P09038; R&D Systems), N2 (17502048; Thermo), nonessential amino acids (cat. no. 1114005; Thermo), and β-mercaptoethanol (cat. no. M6250; Sigma, St. Louis, MO, USA) in Dulbecco’s modified Eagle’s medium/F12 medium (cat. no. 11330032; Gibco) for 5 days. Finally, mature neuronal cells were induced to differentiate from the neuronal precursor cell differentiation media using B27 (17504044; Gibco) instead of bFGF for approximately 10 days.

For the generation of cardiomyocytes, hiPSCs were differentiated into mesodermal progenitor cells using the glycogen synthase kinase-3 inhibitor CHIR99021 (cat. no. 4423; Tocris) in Matrigel-coated dishes (cat. no. 354230; Corning), and the mesodermal progenitor cells produced were further differentiated into a cardiac mesodermal lineage using Wnt-59 (cat. no. S7037; Selleckchem). Cardiac progenitor cells were induced from mesodermal lineage cells via B-27 supplementation (cat. no. 17504–044; Gibco). Upon the observation of contracting cardiomyocytes in the plates, the beating cells were purified with sodium lactate as previously described^41,42^.

For hepatocyte-like cells, we used a previously described differentiation protocol^43,44^ with some modifications. hiPSCs were plated in Matrigel-coated dishes (cat. no. 354277; Corning) 1 day before the initiation of endodermal differentiation by treatment with Activin A (cat. no. 120–14E; Peprotech), sodium butyrate (cat. no. B5887; Sigma), and CHIR99021 (cat. no. SML1046; Sigma). Subsequent to the formation of definitive endodermal cells, bone morphogenic protein 4 (cat. no. 120–05ET; Peprotech) and SB431542 (cat. no. 1614; Tocris) were added to differentiate the hepatic endodermal linage. Finally, hepatocyte-like cells were induced by treatment with fibroblast growth factor 4 (cat. no. 100–31; Peprotech), hepatocyte growth factor (cat. no. 100–39; Peprotech), and oncostatin M (cat. no. 300–10; Peprotech) with dexamethasone (cat. no. D4902; Sigma).

### Sample preparation

Genomic DNA (gDNA) and RNA samples for genetic stability tests were prepared from collected cell pellets using a DNeasy Blood & Tissue kit (cat. no. 69504; Qiagen, Valencia, CA, USA) and an RNeasy plus mini kit (cat. no. 74136; Qiagen).

### Karyotyping

The G-banded karyotypes of 20 single clones of each cell line in metaphase with a band resolution of 500 were analysed.

### Mycoplasma test

Supernatants from PSC culture medium were used for mycoplasma tests. Nested PCR targeting 12 representative mycoplasma samples was performed using a TaKaRa PCR Mycoplasma Detection Set (TaKaRa, Shiga, Japan) according to the manufacturer’s instructions.

### Immunocytochemistry

Cells were fixed with 4% paraformaldehyde and stained with antibodies targeting protein markers of the specific lineages of cells [anti-doublecortin (cat. no. sc-8600; Santa Cruz Biotechnology, Santa Cruz, CA, USA), anti-Tuj1 (cat. no. A-27023; Thermo-Fisher), and anti-microtubule-associated protein 2 (cat. no. T8578; Sigma, St. Louis, MO, USA)]. Fluorescently tagged secondary antibodies were then used to detect the proteins. 4ʹ, 6-Diamidino-2-phenylindole was used for the counterstaining of nuclei.

### Fluorescence-assisted cell sorting analysis

Cells were dissociated for 10 min using 1× TrypLE Express (Thermo Fisher) and washed with phosphate-buffered saline (PBS) containing 1% foetal bovine serum (FBS). For intracellular marker staining, cells were fixed, permeabilized with BD Cytofix/Cytoperm solution (BD Biosciences), and stained with 1 μg of anti-SOX17 (R&D Systems), anti-ALB (Dako), and anti-HNF4A (Santa Cruz Biotechnology) antibodies. For surface marker staining, cells were fixed in 4% paraformaldehyde (Sigma-Aldrich) for 20 min at room temperature, washed with PBS containing 1% FBS and then incubated for 30 min at 4 °C with 1 μg of anti-CXCR4 (BD Biosciences) and anti-asialoglycoprotein receptor 1 (Santa Cruz Biotechnology) antibodies. Flow cytometry was performed using a BD FACSCalibur instrument (BD Biosciences).

### Single-nucleotide polymorphism (SNP) chip data processing for single-nucleotide variant (SNV) and copy number variation (CNV) analyses

SNP genotyping was performed using an Affymetrix CytoScan HD array (Affymetrix), which interrogates 2.6 million markers, including 750,000 SNPs and 1.9 million non-polymorphic probes, across the human genome. Genomic DNA (1 µg input) was amplified and labelled according to the manufacturer’s protocols.

For SNV analysis, probe calls were extracted to compare the differences between samples. An in-house script based on MySQL was applied to calculate the changes between the control (hiPSC lines) and case (differentiated cell lines) samples. Those SNVs showing differentiation between the control and case samples were subjected to ANNOVAR analysis^45^ for annotation from Ensembl identifiers to HGNC gene symbols. To identify rare variants with allele frequencies of less than 0.1 in the general population and the Korean population, we also performed annotations with reference to the 1000 Genomes^46^ and KRG databases (http://152.99.75.168/KRGDB). Thereafter, we evaluated the correlations between nonsynonymous SNVs and gene expression levels.

For CNV analysis, the raw data were analysed using the Chromosome Analysis Suite (ChAS) v3.2 (Affymetrix). For QC, the median absolute pairwise difference score, measuring the variability in the log2 ratio, was set to ≤ 0.25, and SNP-QC, measuring how well genotype alleles were resolved in the microarray data, was set to ≥ 15. In addition, the waviness standard deviation was set to ≤ 0.12 according to the manufacturer’s recommendations. CNV segments of more than 100 kbp and 25 marker counts were considered. In the present study, we only considered CNVs within exonic regions, and we used the UCSC (hg19) database to identify genes within CNV areas. Moreover, CNVs were detected based on weighted log2 ratios and allelic differences. Manual inspection was applied to filter out false positives. All CNVs spanning centromeric regions and those on the X and Y chromosomes were considered false positives and were thus excluded.

### eSNP karyotyping using RNA-seq data

eSNP karyotyping was performed as previously described^17^. SNPs were called using GATK HaplotypeCaller^47^ after the raw RNAseq reads were aligned to the human reference genome (GRCh 38) using TopHat2^48^. SNPs with a minimal minor allele frequency in the total allele depth of less than 0.2 and a low read depth (below 20 reads) were discarded. For visualization, the moving median values for allelic ratios (major to minor) were plotted along the chromosome positions using a window of 151 SNPs.

### Transcriptome profiling

In total, 22 samples were analysed using RNA sequencing to investigate the differential expression of genes between hiPSCs and differentiated cell lines and to examine differentiation processes at the transcriptome level. mRNA-seq libraries were prepared using an Illumina TruSeq RNA Sample Preparation Kit (Illumina). We sequenced 100-nt paired-end stranded reads in an Illumina HiSeq 2500 system (Illumina) according to the manufacturer’s protocols. Count-based transcriptome analysis was performed^49^. The reads were mapped to the reference human genome (GRCh37, hg19) using the STAR 2.5.2b aligner^50^. Alignment was performed using a 2-pass approach by applying the splice junctions detected in the first alignment run to the second alignment. A splice junction database was constructed from the Ensembl database (GRCh 37.73, hg19). After the quantification of gene-based expression counts using htseq-count software, the read counts were normalized via relative log expression (RLE) using the DESeq2 package^51^. After the estimation of dispersion, we calculated the differential expression of genes based on negative binomial tests. A gene was considered to be differentially expressed when the false discovery rate (FDR) value was less than 0.01 and the |log_2_ fold change| was ≥ 4 between differentiated cell lines versus the original hiPSCs. BiomaRt packages^52^ were applied to obtain the corresponding HUGO Gene Nomenclature (HGNC) symbols from the Ensembl Gene identifier. Differentially expressed genes (DEGs) with HGNC gene symbols were considered for downstream analysis. To calculate changes in the expression of tumourigenicity-related genes, we evaluated 707 cancer-related genes^20,21^.

Having converted the raw count values for each gene using regularized log transformation, a sample distance matrix was constructed using the DESeq2 package^51^ Principal component analysis (PCA) was performed using an in-house script based on a dedicated DESeq2 function. To determine the functional enrichment of significant DEGs, gene ontology (GO) analysis was carried out in conjunction with statistical tests based on biological processes using GeneAnswers^53^. The top five GO categories with an FDR of less than 0.05 were considered for assessment.

### SNV calling using RNA-seq data

After alignment to the human reference genome as described above, the reads were processed by using the best practice pipeline, which includes the replacement of read groups, marking of duplicate reads, base recalibration, and realignment of insertions/deletions (indels) with GATK 4.0.4.0. Thereafter, variant calling was performed using GATK HaplotypeCaller. Variant filtration was performed based on the following criteria: ReadPosRankSum < −2.0, MQRankSum < −2.0, QUAL < 30.0, QD < 3.0, FS> 30.0, MQ < 30.0, DP < 10, and GQ < 10.0. The resulting SNPs were annotated using snpEff^54^ with the 1000 Genomes^46^ and Korean Reference Genome (KRG) 1100 databases (http://152.99.75.168/KRGDB).

### HLA typing using RNA-seq data

HLA calling of the RNA-seq data was implemented to identify rare and common HLA alleles in both the hiPSCs and their derivatives using HLAProfiler^55^. After the assembly of a database as a reference, HLA calling was performed based on the developer’s recommendations. The results included major HLA genes for two alleles to identify the homozygous HLA type. The prediction of HLA type was performed when a sample exhibited more than 100 reads of the *HLA* gene. The final HLA type for each cell line was inferred based on Proportion_reads, Proportion_signal, Correlation, error, and Final_score values.

### Time-course analysis

To investigate dynamic expression changes during differentiation into the three germ layers, the count-based raw values of each gene for all 22 samples were used as the initial materials. After normalization using estimated size factors with the DESeq2 package^51^, the expression values were transformed using the log 2 function. Differentiation-related expression dynamics were calculated using TimesVector v1.03^56^. The K-value, which is the number of clusters targeted for detection, was evaluated using the following equation:

1 $${\rm{K}}=-\,85.71+28.57{\rm{x}},$$

where x is the product of the number of conditions and time points. The K-value was adjusted according to the data characteristics following the developer’s recommendations. TimesVector was applied to groups of cells that showed distinct expression patterns or similar expression patterns under K = 85. After manual filtering to remove false-positive gene clusters, we identified significant gene clusters showing dynamic expression during differentiation. We also used the BiomaRt package for gene annotation^52^.

### Variant calling using WES

WES was performed on genomic DNA from hiPSCs and DE cells at passage 20 and hiPSCs, DE cells, HE cells, and HLCs at passage 37 to identify SNPs and short indels transcribed into RNA. The exome region was captured using a SureSelect V6+UTR kit (Agilent) and sequenced on a NovaSeq 6000 system (Illumina). After mapping against the GRCh38/hg38 assembly using BWA^57^, variant calling was performed using the GATK v4.0.4.0 tool^58^, including the marking of duplicates, base recalibration, and indel realignment. Joint variant calling was then performed with all samples using GATK HaplotypeCaller. Variant and gene annotation was performed with SnpEff^54^ and dbSNP151^59^. Among the 132,248 called variants, the selection of variants for further analysis was performed as follows: (1) candidate mutations present in the control cell lines were discarded; (2) candidate mutant alleles with an allele frequency of greater than 20% were selected; (3) candidate mutations annotated in protein-coding regions were selected; and (4) candidate mutations annotated as intron variants and up-/downstream variants were discarded. In total, 827 variants were finally selected for downstream analysis.

### Statistics

All data are presented as the mean + SEM. Differences between two means were analysed using Student’s t-test.

### Cell lines and data availability

Raw data from the SNP chip and RNA sequencing analyses performed in this study have been deposited in the Clinical & Omics Data Archive (CODA, http://coda.nih.go.kr) under accession numbers R001856 and R001855, respectively. The cell lines used in this study are also available through the Korea Stem Cell Bank Institute (http://kscr.nih.go.kr).

## Supplementary information

Supplementary Information.

Suppementary Table S2.

Suppementary Table S3.

Suppementary Table S4.

Suppementary Table S5.

Suppementary Table S6.

Suppementary Table S7.

Suppementary Table S8.

Suppementary Table S9.

Suppementary Table S10.
