# Supplementary material for: Development of genetic quality tests for good manufacturing practice-compliant induced pluripotent stem cells and their derivatives
Source: Sci Rep. 2020 Mar 3;10:3939. doi: 10.1038/s41598-020-60466-9 (PMC7054319; doi:10.1038/s41598-020-60466-9)
Supplement: Supplementary file 1 — Supplementary Information. [file 41598_2020_60466_MOESM1_ESM.docx]

**Supplementary Information**

**Development of genetic quality tests for** **good manufacturing practice-compliant induced pluripotent stem cells and their derivatives**

Hye-Yeong Jo^1,2^, Hyo-Won Han^1^, Inuk Jung^3^, Ji Hyeon Ju^4^, Soon-Jung Park^5^, Sunghwan Moon^5^, Dongho Geum^6^, Hyemin Kim^7^, Han-Jin Park^7^, Sun Kim^2,3^, Glyn N Stacey^8,9,10^, Soo Kyung Koo^1^, Mi-Hyun Park^1*^, Jung-Hyun Kim^1*^

^1^Division of Intractable Diseases, Center for Biomedical Sciences, Korea National Institute of Health, Cheongju, South Korea

^2^Interdisciplinary Program in Bioinformatics, Seoul National University, Seoul, South Korea

^3^Bioinformatics Institute, Seoul National University, Seoul, South Korea

^4^Division of Rheumatology, Seoul St. Mary's Hospital, College of Medicine, Catholic University of Korea, Seoul, South Korea

^5^Department of Medical Science, Konkuk University School of Medicine, Seoul, South Korea

^6^Department of Medical Science, Medical School, Korea University, Seoul, South Korea

^7^Department of Predictive Toxicology, Korea Institute of Toxicology, Daejeon, South Korea

^8^International Stem Cell Banking Initiative, 2 High St, Barley, Hertfordshire, SG88HZ, UK

^9^National Stem Cell Resource Center, Institute of Zoology, Chinese Academy of Sciences, Beijing 100190, China

^10^Innovation Academy for Stem Cell and Regeneration, Chinese Academy of Sciences, Beijing 100101, China

***Corresponding authors**

Jung-Hyun Kim, Ph.D., Division of Intractable diseases, Korea National Institute of Health, Osongsaengmyeong2-ro 202, Cheong-Ju, 28160, South Korea

Tel:+43-249-2512

E-mail: kjhcorea@korea.kr

Mi-Hyun Park, Ph.D., Division of Intractable Diseases, Korea National Institute of Health, Osongsaengmyeong2-ro 202, Cheong-Ju, 28160, South Korea. Tel:+43-249-2514

E-mail: mihyun4868@korea.kr


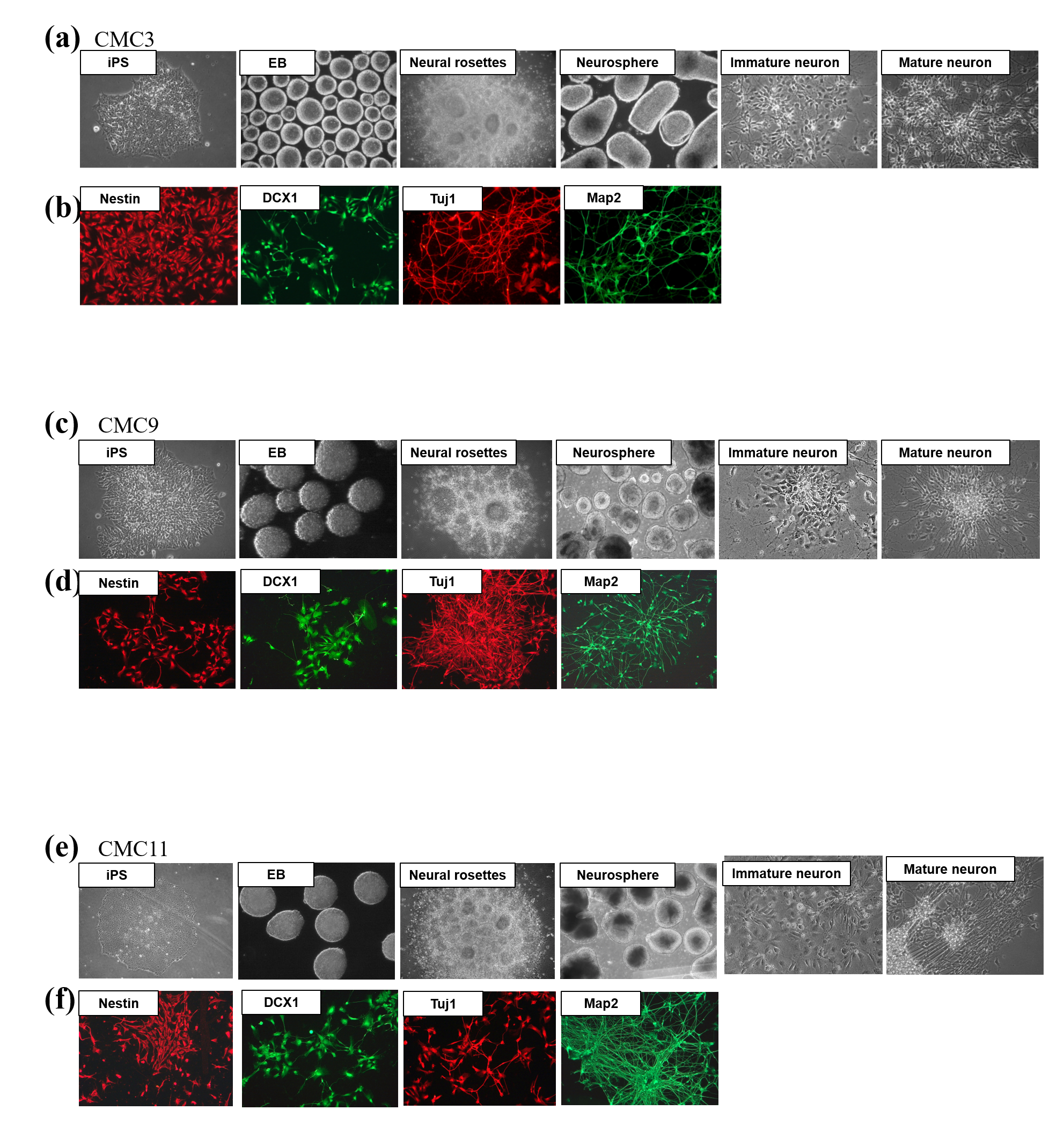


**Supplementary Figure S1. Differentiation of homozygous human induced pluripotent stem cell (hiPSC) lines into the ectodermal lineage (neuronal cells).**

Neuronal cell differentiation from the examined hiPSC lines. The (**a, b**) CMC3, (**c, d**) CMC9, and (**e, f**) CMC11 lines differentiated into neuronal cells. (**a, c, e**) Representative images of cell morphology during differentiation. (**b, d, f**) Fluorescence images of the neuronal cell markers nestin, doublecortin (DCX1), Tuj-1, and microtubule-associated protein 2 (MAP2) (×100 magnification).


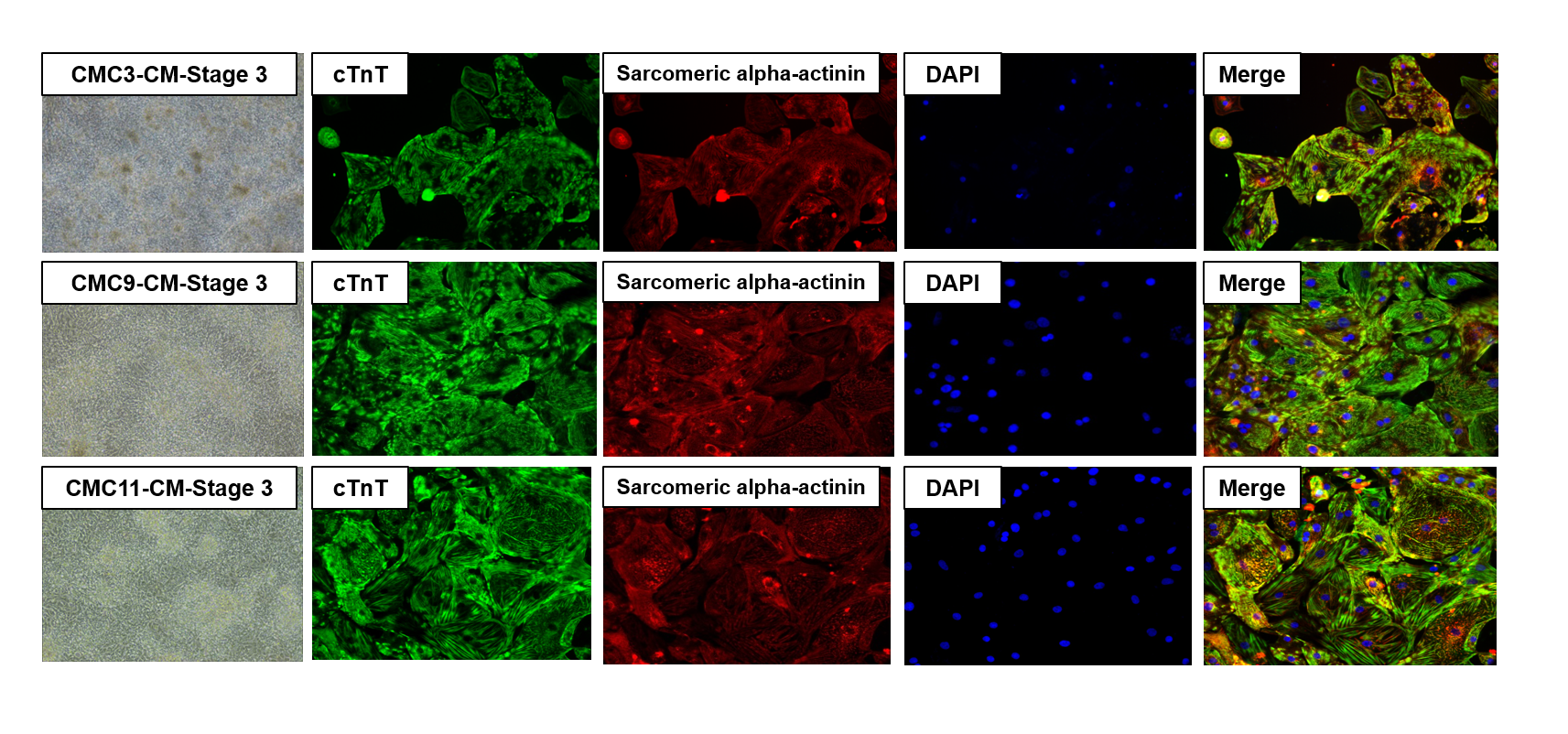


**Supplementary Figure S2. Differentiation of homozygous human induced pluripotent stem cell (hiPSC) lines into the mesodermal lineage (cardiomyocytes).**

Cardiomyocyte differentiation from the examined hiPSC lines. Representative fluorescence images of the cardiomyocyte markers cardiac troponin T (cTnT) and sarcomeric α-actinin. Bright-field images are shown for the final stage of differentiation of the CMC3, CMC9, and CMC11 lines into cardiomyocytes (×200 magnification)


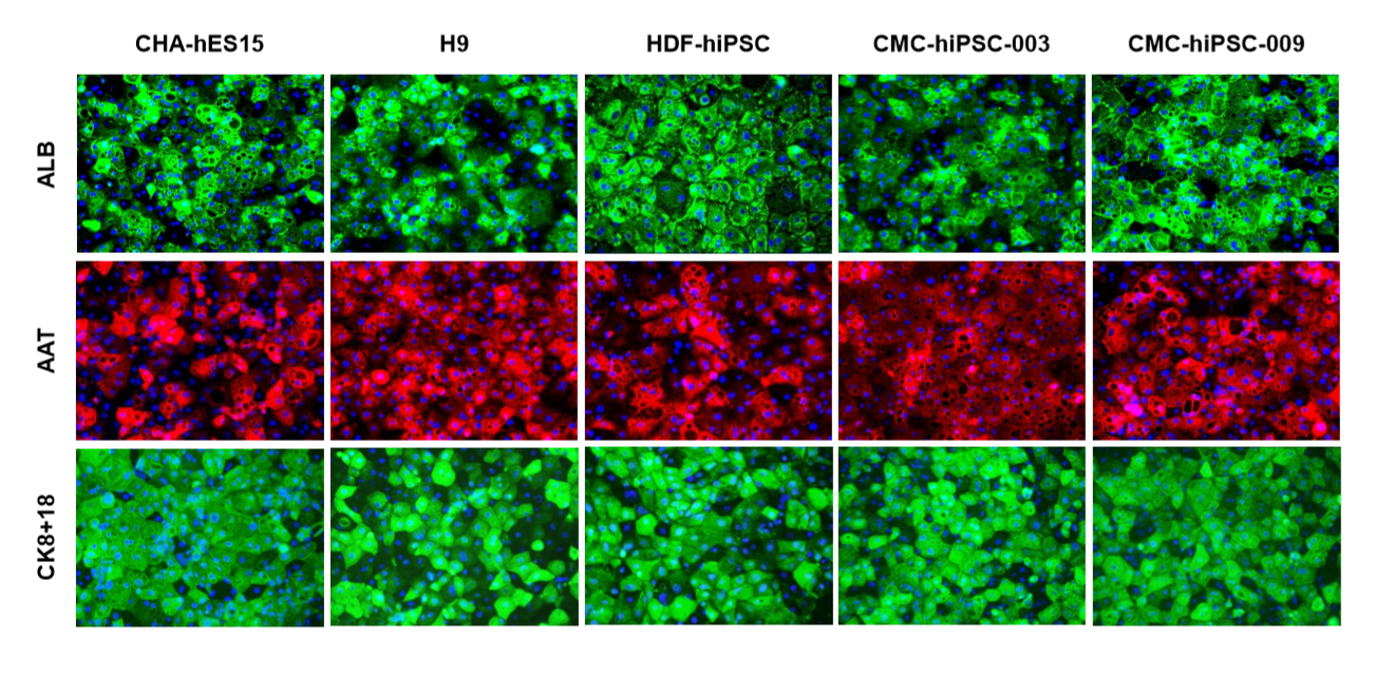


**Supplementary Figure S3. Differentiation of homozygous human induced pluripotent stem cell (hiPSC) lines into the endodermal lineage (hepatocytes)**

Hepatocyte-like cell differentiation from the examined hiPSC lines. Representative images of hepatocyte-like cell markers (ALB, AAT, and CK8^+^18 proteins; ×200 magnification). H9 and human dermal fibroblast-derived hiPSC lines were used as positive control cell lines. The final differentiated cells were used for immunocytochemical staining.


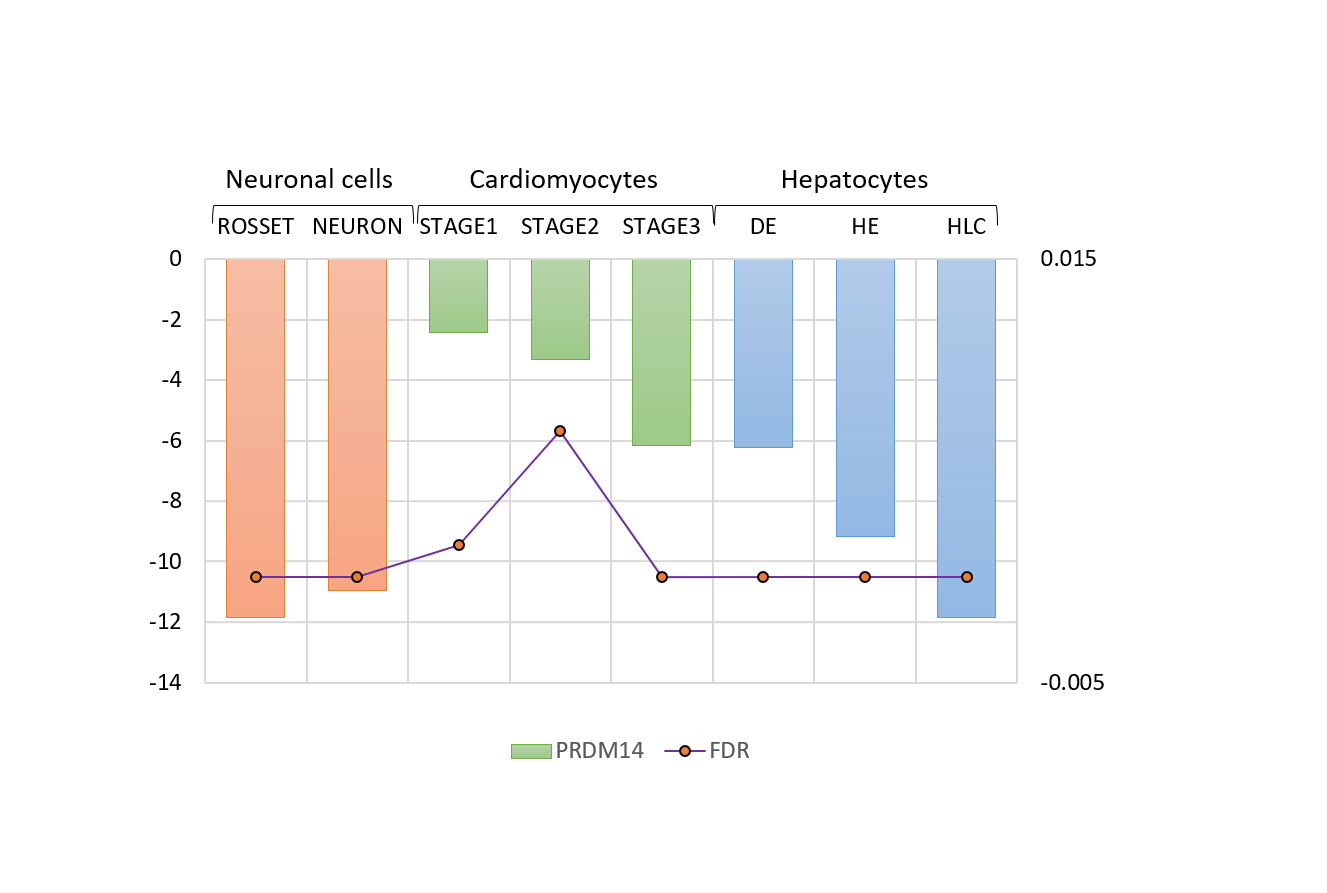


**Supplementary Figure S4. Expression level of PRDM14 at each stage of differentiation.**

The expression values of PRDM14 in the differentiated cells compared with those in the original human induced pluripotent stem cells (hiPSCs) are shown at each stage of differentiation: neuronal cells (orange bars), cardiomyocytes (green bars), and hepatocytes (blue bars). Purple bars indicate false discovery rate (FDR) values.


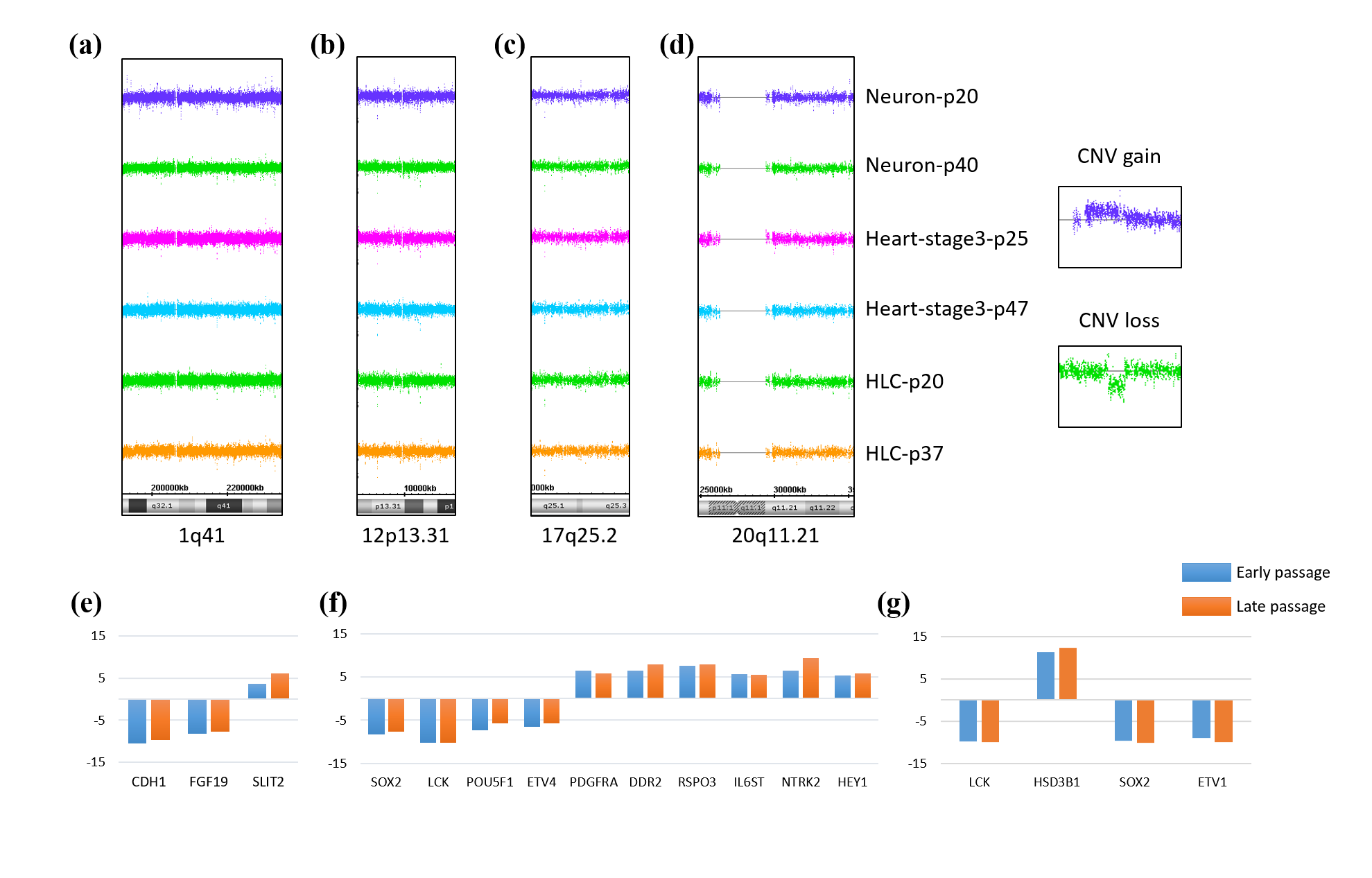


**Supplementary Figure S5. Identification of genomic stability in human induced pluripotent stem cell (hiPSC) lines and differentiated cell lines.** Recurrent copy number variants (CNVs) in 1q41, 12p13.31, 17q25.2, and 20q11.21 at the final stage of differentiation are shown in **a, b, c,** and **d**, respectively. The signal intensities of neuronal cells at passages 20 and 40, stage 3 cardiomyocytes at passages 25 and 47, and hepatocyte-like cells (HLCs) at passages 20 and 37 are specified in order. Significant differentially expressed cancer-associated genes in neuronal cells (**e)**, cardiomyocytes (**f**), and HLCs (**g**) were also identified to evaluate the genomic stability of the cell lines at early (blue bars) and late (orange bars) passages.


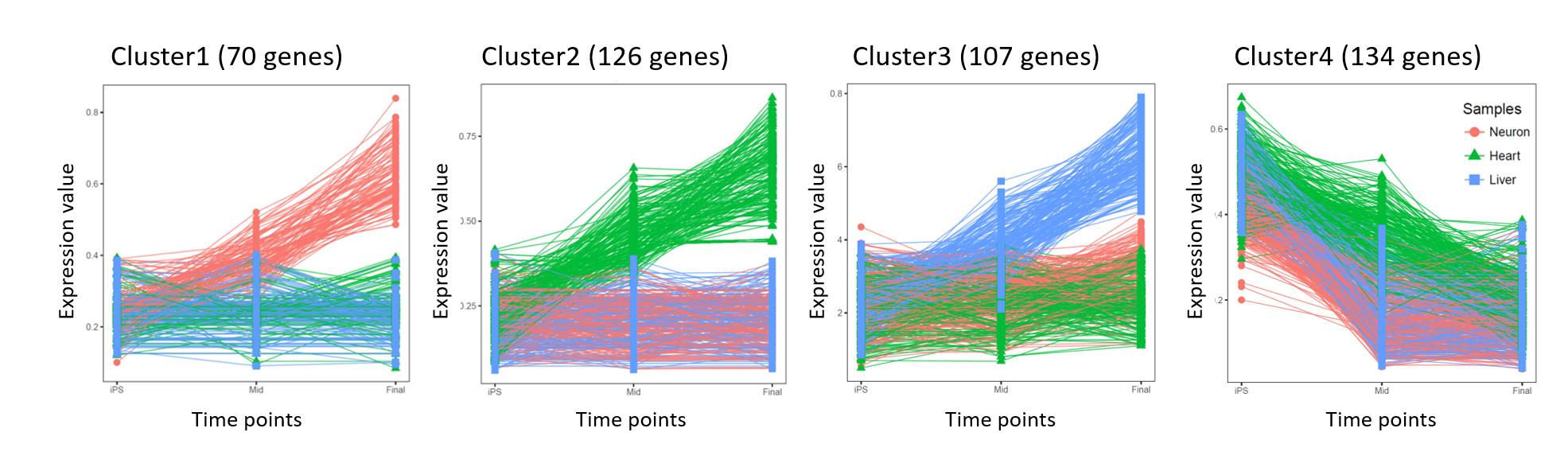


**Supplementary Figure S6. Dynamic expression patterns in sequential differentiation stages**

Lineage-specific gene clusters are shown. Neuronal cells, cardiomyocytes, hepatocyte-like cells, and human induced pluripotent stem cell (hiPSC)-specific clusters are indicated as clusters 1, 2, 3, and 4, respectively. Expression patterns during differentiation into neuronal cells (orange bars), cardiomyocytes (green bars), and hepatocyte-like cells (blue bars), specified with the number of genes for each cluster.

**Supplementary Tables**

**Supplementary Table S1. Characteristics of the three distributed lines.**

|  | Origin | Derivation method | HLA type (*A*B*DRB1) | Frequency Ranking |
| --- | --- | --- | --- | --- |
| CMC-hiPSC-003 | Bone marrow | Sendi-Virus | *33:03 *44:03 *13:02 | 1 |
| CMC-hiPSC-009 | Cord blood cell | Sendi-Virus | *24:02 *07:02 *01:01 | 2 |
| CMC-hiPSC-0011 | Cord blood cell | Sendi-Virus | *11:01 *15:01 *04:06 | 7 |

**Supplementary Table S2. (Related to Table 1) The number of SNPs maintained during differentiation.** Excel file available.

**Supplementary Table S3. (Related to Table 1) The number of SNPs maintained under prolonged culture.** Excel file available.

**Supplementary Table S4. (Related to Table 1 and Supplementary Fig. S4).** Single-nucleotide variants (SNVs) in differentiated lines and correlations with gene expression levels. Excel file available.

**Supplementary Table S5. (Related to Table 2).** Human leucocyte antigen (HLA) types of human induced pluripotent stem cells (hiPSCs) and differentiated cells. Excel file available.

**Supplementary Table S6. (Related to Fig. 3F).** List of differentially expressed genes in neural progenitor rosettes and neuronal cells compared with the original human induced pluripotent stem cells (hiPSCs). Top-ranked upregulated genes are indicated in sky blue. Excel file available.

**Supplementary Table S7. (Related to Fig. 3G).** List of differentially expressed genes in stages 2 and 3 of cardiomyocyte differentiation compared with the original human induced pluripotent stem cells (hiPSCs). Top-ranked upregulated genes are indicated in sky blue. Excel file available.

**Supplementary Table S8. (Related to Fig. 3H).** List of differentially expressed genes in definitive endoderm (DE) cells and hepatocyte-like cells (HLCs) compared with the original human induced pluripotent stem cells (hiPSCs). Top-ranked upregulated genes are indicated in sky blue. Excel file available.

**Supplementary Table S9. (Related to Figs. 4 and 5).** Lists of genes in clusters 38 and 71. The Ensemble ID, official gene symbol, Entrez ID, and gene name are included. Excel file available.

**Supplementary Table S10. (Related to Supplementary Fig. S6).** Complete list of genes in clusters 1, 2, 3, and 4. Excel file available.
